# Genetic Variation Analysis and Research on Biological Characteristics of Duck Hepatitis Virus Type 3: A Comparison Between Historical Strains in Yunnan and Recent Epidemic Strains

**DOI:** 10.3390/vetsci12100923

**Published:** 2025-09-23

**Authors:** Sixian Lan, Aiguo Xin, Ke Li, Zhengju Yuan, Rong Zhao, Zhishun Chang, Wengui Li, Hongya Yan

**Affiliations:** 1 College of Veterinary Medicine, Yunnan Agricultural University, Kunming 650201, China; lsx08180818@163.com; 2 Institute of Poultry Science, Yunnan Academy of Animal Science and Veterinary Medicine, Kunming 650224, China; aiguo_xin@hotmail.com (A.X.); johnsonlike@163.com (K.L.); 13987103693@163.com (Z.Y.); zrtl_510@163.com (R.Z.); changzhishun99@163.com (Z.C.)

**Keywords:** duck hepatitis A virus type 3 (DHAV-3), Yunnan isolates, genetic variation, pathogenicity

## Abstract

Duck viral hepatitis, caused by Duck Hepatitis A Virus Type 3 (DHAV-3), remains a major threat to young ducklings. Although DHAV-3 has circulated in China since the 1999s, the complete genomic architecture, exact virulence parameters, and evolutionary distance between early Yunnan isolates and current field strains have remained undefined. This study investigated six DHAV-3 strains isolated in Yunnan Province, China, between 2004 and 2006, to elucidate their genetic and biological characteristics. Full-genome sequencing and phylogenetic analysis revealed >99.5% nucleotide and >99.6% amino acid identity among the strains, suggesting a common ancestral origin. In vivo challenge assays showed rapid onset of clinical signs and >90% mortality in ducklings within 36 h post-inoculation. Embryonic deaths began at 24 h post-infection and peaked by 90 h. Viral replication was efficient in DEF, DEK, Vero, and BHK-21 cells, but absent in chicken fibroblasts (DF-1). Comparative genomic analysis between the YN/LR/2005 strain and recent field isolates (2022–2024) revealed substantial nucleotide divergence in structural regions, with 32 unique amino acid substitutions—all five located in the immunodominant VP1 region that may influence viral antigenicity and host interaction—alongside changes in N-glycosylation sites and alterations in protein secondary structure. Histopathological examination confirmed characteristic hepatic lesions. These findings demonstrate that while DHAV-3 has undergone genetic evolution, it retains high virulence, underscoring the need for ongoing molecular surveillance and supporting future vaccine and diagnostic development.

## Simple Summary

Duck hepatitis A virus type 3 (DHAV-3) is a highly contagious pathogen that causes severe liver damage and rapid mortality in ducklings. To understand its evolutionary trends in Yunnan Province, China, we analyzed six historical DHAV-3 strains collected between 2004 and 2006. Their genetic characteristics, replication efficiency in various cell lines, and pathogenicity in embryos and ducklings were systematically evaluated. All strains exhibited over 99.5% nucleotide identity, indicating a shared origin. Infection led to early clinical signs and high mortality within 36 h. While replication was limited in chicken fibroblasts (DF-1), robust viral growth and cytopathic effects were observed in duck embryo fibroblasts (DEF), duck embryo kidney cells (DEK), Vero (African green monkey kidney), and BHK-21 (baby hamster kidney) cells. Compared with strains circulating after 2022, the 2005 strain displayed distinct mutations in structural genes, altered glycosylation patterns, and shifts in protein secondary structure. Histopathological analysis confirmed severe hepatic lesions. These findings highlight the genetic stability and sustained virulence of DHAV-3, offering critical insights for improved diagnostics, vaccine development, and disease control. Importantly, the identified mutational signature provides a molecular barcode for retrospective screening and prospective early-warning surveillance of DHAV-3 outbreaks. Notably, the Yunnan 2005 lineage represents a pivotal “ancestral node” that bridges historical epidemics and recent epidemics, making it a key reference for tracing global DHAV-3 transmission routes.

## 1. Introduction

Duck viral hepatitis (DVH) is an acute and highly fatal infectious disease caused by the duck hepatitis A virus (DHAV). It is characterized by rapid onset, swift transmission, a short disease clinical course, and high mortality rates [1,2]. Clinically, infected ducklings primarily exhibit neurological symptoms, including convulsions, seizures, and opisthotonos. Pathological findings typically include hepatomegaly, nephromegaly, hemorrhage, gallbladder swelling, and splenomegaly [3,4,5]. The disease predominantly affects ducklings younger than 35 days, with the highest susceptibility observed in those under 7 days of age. Mortality generally occurs within 24–72 h after symptom onset, with younger ducklings experiencing higher fatality rates. Adult ducks often show subclinical infections [6]; however, the virus can persist in the rearing environment and be vertically transmitted from breeding ducks to their offspring [7].

Duck hepatitis A virus (DHAV) is a highly pathogenic agent responsible for duck viral hepatitis (DVH), an acute and often fatal disease affecting ducklings worldwide. Based on phylogenetic and serological analyses, DHAV has been classified into three antigenically distinct genotypes: DHAV-1, DHAV-2, and DHAV-3, with minimal cross-protection observed among them [8,9,10,11,12,13]. DHAV-3 and DHAV-1 are assigned to separate genogroups within the same Picornaviridae species [11] and display congruent genome architectures. The principal genetic divergence between the two viruses resides in the lengths of VP1, the 5′ UTR and the 3′ UTR, all of which are extended in DHAV-3, resulting in a correspondingly longer complete genome. Notably, duck astrovirus types 1 and 2 (DAstV-1 and DAstV-2) have been identified as causative agents of DVH types II and III, respectively [14,15]. The DHAV-3 genome is approximately 7.8 kb in length and comprises a single open reading frame encoding a polyprotein with a genomic structure arranged as 5′UTR-L-VP0-VP3-VP1-2A (2A1-2A2-2A3)-2B-2C-3A-3B-3C-3D-3′UTR. This ORF encodes three capsid proteins (VP0, VP1, and VP3) and nine non-structural proteins (2A1, 2A2, 2A3, 2B, 2C, 3A, 3B, 3C, and 3D) [16,17]. VP1, as the primary surface protein and core antigenic structure of DHAV, plays a crucial role in viral infection. It serves not only as the main target for neutralizing antibodies, directly inducing their production in host animals, but also as the primary region for viral antigenic variation [18,19]. Genetic mutations in VP1 alter the virus’s antigenic properties, enabling it to evade immune recognition by neutralizing antibodies within the host. This leads to immune escape and the emergence of new epidemic strains. This process is also a major cause of small RNA virus vaccine failure [18,19]. Historically, DHAV-1 was the dominant genotype causing outbreaks in China; however, recent epidemiological data indicate that DHAV-3 has become the predominant genotype circulating across multiple provinces including Beijing, Guangxi, Fujian, Shandong, Jiangsu, Zhejiang, Anhui, Guangdong, Hubei, and Sichuan [20,21,22]. Although a live DHAV-1 vaccine is widely used commercially, a licensed live DHAV-3 vaccine (the HB80 strain) has only been introduced to duck farms in China in 2024, which can provide ≥80% protection [23]. However, the attenuation mechanism, immune protection mechanism, and optimization strategies of the HB80 vaccine strain require further investigation. This study conducted genomic structure analysis, phylogenetic tree construction, and nucleotide/amino acid sequence homology comparisons for six DHAV-3 strains preserved in the laboratory. It further performed homology comparisons of genomic structural regions, N-terminal glycosylation site analysis, protein secondary structure prediction, amino acid difference site analysis, and histopathological observations of duckling tissues. The objectives were to elucidate the genetic variation between historical Yunnan DHAV-3 strains and currently circulating strains, provide scientific data for regional viral evolution of DHAV-3, and offer reference for vaccine strain selection against DHAV-3.

## 2. Materials and Methods

### 2.1. Animals Ethics Statement

All experimental procedures involving animals were reviewed and approved by the Animal Welfare and Ethics Committee of the Yunnan Academy of Animal Science and Veterinary Medicine (approval no. YNASVI01-2025015). All protocols were conducted in strict compliance with national guidelines and regulations for laboratory animal welfare. No notifiable epizootic diseases were involved in this study. Every effort was made to minimize animal suffering and distress throughout the experiments.

### 2.2. Viruses and Main Materials

#### 2.2.1. Virus Isolates and Cell Lines

Six DHAV-3 isolates were recovered from deceased sick ducks on duck farms in Yunnan Province between 2004 and 2006 by our laboratory. The isolates have been preserved at −80 °C in ultra-low temperature freezers (Anhui Zhongke Duling Commercial Appliances Co., Ltd. (Hefei, China)). The African green monkey kidney epithelial cell line (Vero), Syrian hamster kidney fibroblast cell line (BHK-21), and chicken embryo fibroblast cell line (DF-1) were maintained in our laboratory. Primary duck embryo fibroblast (DEF), duck embryo kidney (DEK), and duck embryo liver (DEL) cells were prepared in-house following standard protocols.

#### 2.2.2. Animals

The duck embryos and ducklings used in this study were sourced from a healthy core flock of Jianshui Yellow-brown Ducks. Breeding ducks tested negative for both DHAV-1 and DHAV-3 antibodies via indirect ELISA (serologically negative, clean level CL). The average initial body weight of ducklings was 90.2 g.

### 2.3. Reagents and Kits

RNA extraction kits, pMD™18-T vectors, and TRIzol reagent were purchased from Takara Bio Inc. (Shiga, Japan). The FastKing cDNA First Strand Synthesis Kit was obtained from TianGen Bio-Chem Technology (Beijing) Co., Ltd. (Beijing, China). PCR reagents, including 2× Rapid Taq Master Mix, were sourced from Nanjing Novazeen Biotechnology Co., Ltd. (Nanjing, China). Agarose was purchased from Shanghai Jierui Biotechnology Co., Ltd. (Shanghai, China), and PCR product purification kits were supplied by Yunnan Chenlv Biotechnology Co., Ltd. (Kunming, China). All other chemical reagents used in this study were of analytical grade and domestically sourced.

### 2.4. Primer Synthesis

The full-genome amplification primers for DHAV-3 were synthesized according to the method previously described in reference [24] (Table 1). All primers were synthesized by Shanghai Jierui Bioengineering Co., Ltd. (Shanghai, China).

### 2.5. Viral Whole-Genome Amplification and Sequence Analysis

Viral RNA was extracted from infected samples using a commercial RNA extraction kit according to the manufacturer’s instructions. Reverse transcription was performed using a cDNA synthesis kit to obtain complementary DNA (cDNA), which served as the template for subsequent PCR amplification. PCR products were analyzed by 1.5% agarose gel electrophoresis, and target fragments were purified using a commercial gel extraction kit. The purified fragments were ligated into the pMD™18-T vector to construct recombinant plasmids, which were submitted to Kunming Qingke Biotechnology Co., Ltd. (Kunming, China). for Sanger sequencing. Sequencing data were assembled using DNAStar version 11.1 software. Sequence similarity analysis was conducted using the BLAST (Sequence similarity searches were performed using the NCBI web-BLAST service snapshot release 2.16.0, accessed November 2024). tool available at the National Center for Biotechnology Information (NCBI). Phylogenetic trees were constructed using the Neighbor-Joining model based on representative DHAV strains reported in recent years. (Table 2). Amino acid sequence alignment and mutation site analysis were performed using BioEdit version 6.1. N-terminal glycosylation site prediction was conducted using the NetNGlyc 1.0 Server (http://www.cbs.dtu.dk/services/NetNGlyc/, accessed on 20 May 2025), and protein secondary structure prediction was carried out using SOPMA (https://npsa-prabi.ibcp.fr/cgi-bin/npsa_automat.pl?page=npsa_sopma.html, accessed on 22 May 2025).

### 2.6. Cell Adaptation Assay

Primary duck embryo fibroblast (DEF), duck embryo kidney (DEK), and duck embryo liver (DEL) cells were prepared from 12-, 21-, and 14-day-old duck embryos, respectively, following previously described protocols [25,26]. The six DHAV-3 isolates were separately inoculated onto DEF, DF-1, DEK, DEL, BHK-21, and Vero cells. Virus adsorption was carried out at 37 °C for 1 h, after which the inoculum was removed, and an appropriate volume of maintenance medium was added to each well or flask. Cultures were monitored daily for cytopathic effects (CPE), and observations were recorded after three consecutive blind passages to assess viral replication and adaptation.

### 2.7. Animal Pathogenicity Assays

For the embryonic pathogenicity test, 84-day-old duck embryos were used. A total of 24 embryos were divided equally into an experimental group (*n* = 12) and a control group (*n* = 12). Embryos in the experimental group were inoculated with 0.1 mL of viral suspension via the allantoic cavity, while those in the control group received an equal volume of sterile physiological saline. Embryonic viability was monitored continuously for 5 days, and mortality was recorded daily.

For the pathogenicity test in ducklings, 70 healthy 7-day-old ducklings confirmed to be free of common pathogens were randomly assigned to seven groups (six experimental groups and one control group), with 10 ducklings per group. Each duckling in the experimental groups was intramuscularly injected with 0.5 mL of the respective viral isolate, while control animals received 0.5 mL of physiological saline. Clinical symptoms and mortality were observed for 7 consecutive days post-inoculation. Ducklings that died during the observation period were immediately necropsied. Liver and other major tissues were collected, fixed in 4% paraformaldehyde, and submitted to Wuhan Seville Biotechnology Co., Ltd. (Wuhan, China). for histopathological analysis.

Survival curves of duck embryos and ducklings were generated with the Kaplan–Meier method and compared by the log-rank test using GraphPad Prism 9.

### 2.8. Viral Virulence Assay

1% penicillin-streptomycin (100 U/mL penicillin and 100 μg/mL streptomycin) was added to the viral suspension and incubated at room temperature for 2 h to neutralize potential contaminants. The treated viral suspension was then serially diluted tenfold from 10^−2^ to 10^−8^ using phosphate-buffered saline (PBS). Each dilution was inoculated into 12-day-old duck embryos (0.15 mL per embryo) via the allantoic cavity and into 7-day-old ducklings (0.5 mL per duckling) via intramuscular injection. For each dilution, 8 embryos and 6 ducklings were used. Embryos and ducklings were monitored twice daily for clinical signs and mortality. Embryos that died within 24 h post-inoculation were excluded from the analysis. The observation period lasted for 5 days, during which mortality data were recorded. Simultaneously, monolayers of susceptible cells were seeded in 96-well plates and inoculated with 0.1 mL of each viral dilution (10^−2^ to 10^−8^) in 16 replicates per dilution. After 96 h of incubation, wells were examined under a microscope, and the number of wells showing cytopathic effects (CPE) was recorded. The 50% embryo lethal dose (ELD_50_), 50% lethal dose in ducklings (LD_50_), and 50% tissue culture infectious dose (TCID_50_) were calculated using the Karber method.

## 3. Results

### 3.1. Genetic Evolution Analysis of Six Virus Strains

#### 3.1.1. Whole-Genome Sequence Amplification, Sequencing, and Assembly

**Figure 1:**
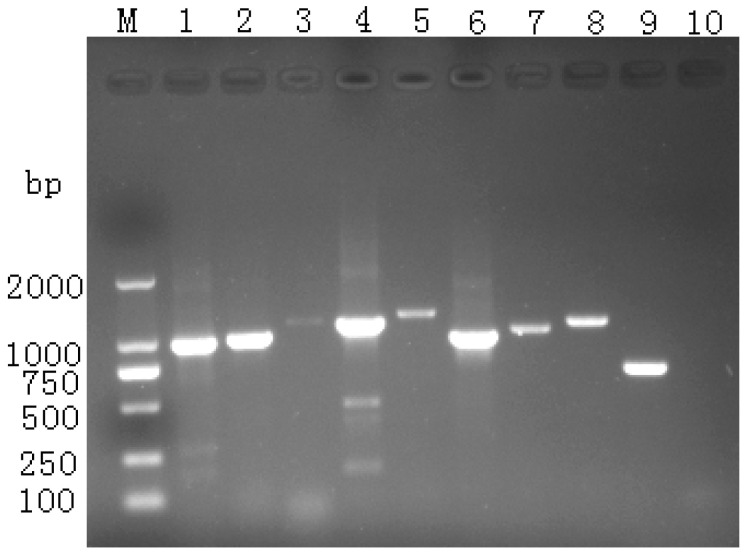
Results of whole-genome sequence segment amplification.

PCR amplification products were analyzed by 1.5% agarose gel electrophoresis, producing bands corresponding to the expected sizes (Figure 1). Following sequencing of the recombinant plasmids, sequence assembly was performed using DNAStar version 11.1 software. The six DHAV-3 isolates were designated as YN/LR/2005, YN/Z/2004, YN/Y/2004, YN/F/2005, YN/K/2005, and YN/21/2006, respectively.

#### 3.1.2. Genome Structure Analysis

The genome length of the YN/LR/2005 strain is 7792 nucleotides (nt), while the YN/K/2005, YN/Z/2004, and YN/Y/2004 strains each possess a genome length of 7791 nt. The YN/F/2005 and YN/21/2006 strains have genome lengths of 7793 nt and 7797 nt, respectively. The overall G+C content of these genomes ranges from 42.87% to 43.08%, with specific values of 43.07%, 43.01%, 43.02%, 43.08%, 43.04%, and 42.87% corresponding to YN/LR/2005, YN/K/2005, YN/Z/2004, YN/Y/2004, YN/F/2005, and YN/21/2006, respectively.

Each genome contains a single open reading frame (ORF) encoding the structural protein P1, as well as the non-structural proteins P2 and P3. These polyproteins are proteolytically cleaved into functional viral proteins, including VP0, VP3, VP1, 2A1, 2A2, 2B, 2C, 3A, 3B, 3C, and 3D. The ORF length is 6756 nt for all strains, flanked by a 5′ untranslated region (UTR) of 651 nt and a 3′ UTR of 366 nt. The poly(A) tail lengths vary slightly among strains, measuring 18, 18, 19, 20, 18, and 24 nt, respectively.

Using the full-length genome sequence of the reference strain C-GY (GenBank accession number: EU352805.2) [27], the genomic structure of each isolate was annotated to determine the relative positions of gene fragments within the complete sequence (Table 3).

#### 3.1.3. Whole-Genome Sequence Homology Analysis

Sequence alignment revealed that the nucleotide sequence identity among all six strains exceeded 99.5%, with amino acid sequence identity above 99.6%. Compared to recently identified strains in China—HNAY2024 (2024) and XY1118 and HNXY23 (2023)—nucleotide sequence identity ranged from 95.5% to 95.7%, while amino acid sequence identity ranged from 98.2% to 98.6%. For strains isolated in 2022, including DZ0918, CF1224, WKX03, and HB-1, nucleotide sequence similarity ranged from 95.3% to 95.9%, and amino acid similarity ranged from 98.3% to 98.6%. The highest nucleotide sequence similarity was observed with the LS strain identified in China in 2014, ranging from 97.1% to 97.3%, with amino acid sequence similarity between 98.8% and 98.9% (Table 4).

#### 3.1.4. Results of Phylogenetic Tree Analysis

**Figure 2:**
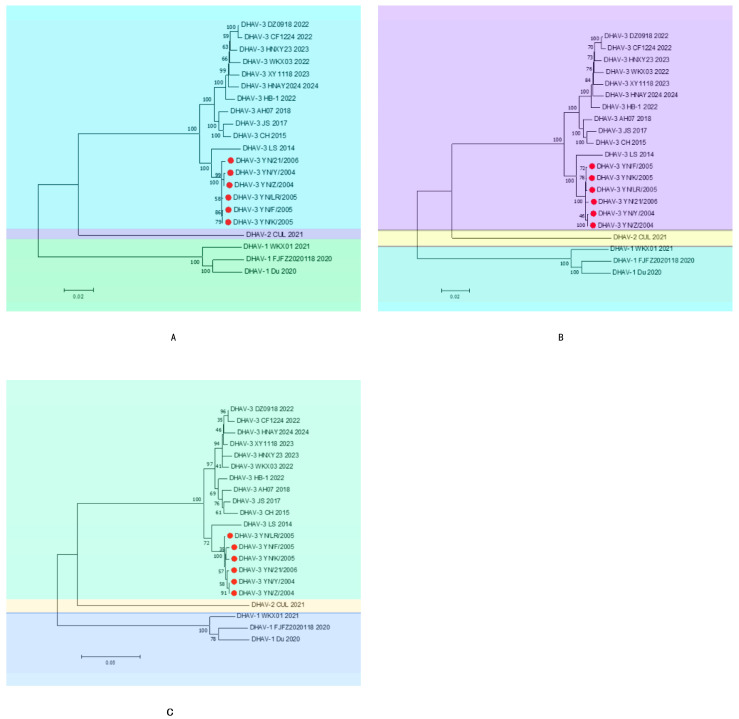
Phylogenetic tree ((**A**): whole genome, (**B**): ORF, (**C**): VP1). The red circles indicate the six strains mentioned in the article.

Phylogenetic analyses based on the whole genome, ORF, and VP1 sequences (Figure 2) demonstrated that all six strains cluster within the same evolutionary branch of DHAV-3, suggesting a common ancestral origin. These strains showed the closest relationship with the DHAV-3 LS strain reported in 2014, grouping within the same evolutionary cluster. They formed distinct subclades alongside the 2018 DHAV-3 AH07, 2017 DHAV-3 JS, and 2015 CH strains within this branch. Although positioned on the same branch as the reference strains from 2022, 2023, and 2024, the six isolates are relatively distant from these recent strains, indicating ongoing genomic variation among DHAV-3 strains in recent years.

### 3.2. Experimental Study on the Pathogenicity and Infectious Characteristics of DHAV-3

#### 3.2.1. Pathogenicity Test on Duck Embryos

**Figure 3:**
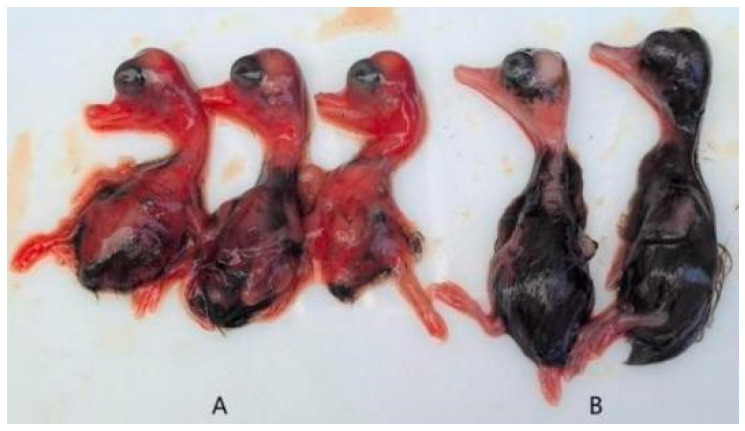
Clinical symptoms of duck embryos. ((**A**): challenge group; (**B**): control group).

**Figure 4:**
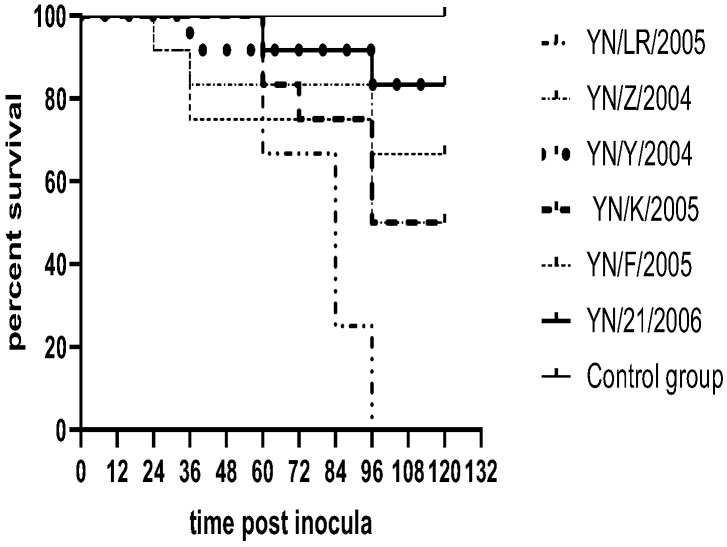
Survival rate of duck embryos.

The pathogenicity assay demonstrated that duck embryos began to exhibit mortality starting at 24 h post-infection, with peak mortality occurring between 60 and 90 h. Deceased embryos displayed developmental retardation accompanied by widespread skin and subcutaneous congestion and hemorrhage. In contrast, embryos in the control group showed normal growth and development without apparent pathological changes (Figure 3). Survival curves for infected duck embryos were generated using the Kaplan–Meier method and plotted with GraphPad Prism 8 (Figure 4).

#### 3.2.2. Pathogenicity Test on Ducklings

**Figure 5:**
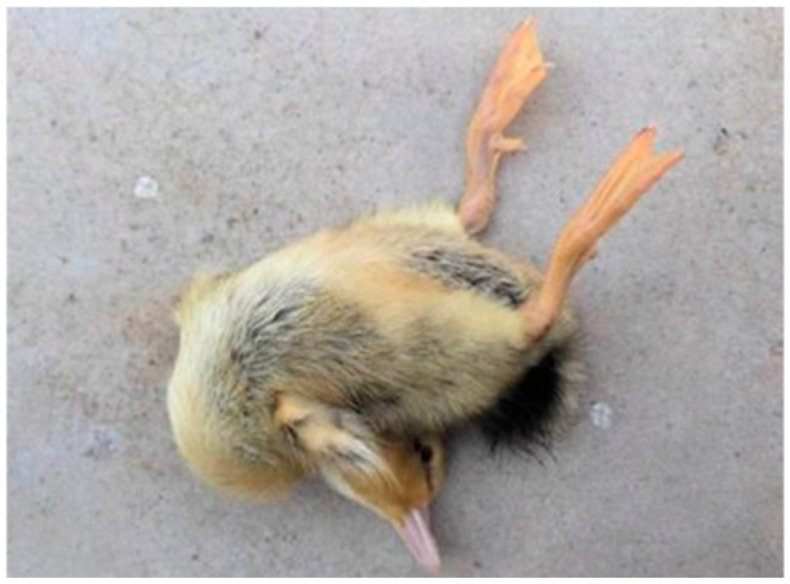
Opisthotonos.

**Figure 6:**
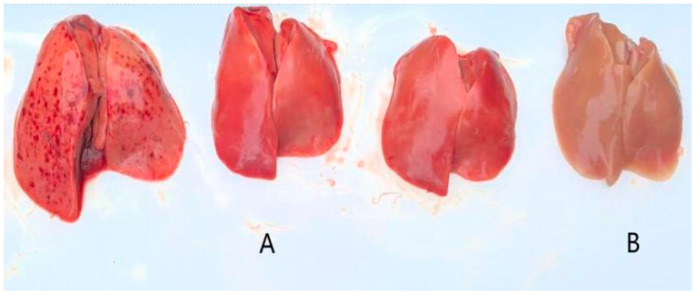
Liver lesions ((**A**): toxic attack; (**B**): control).

**Figure 7:**
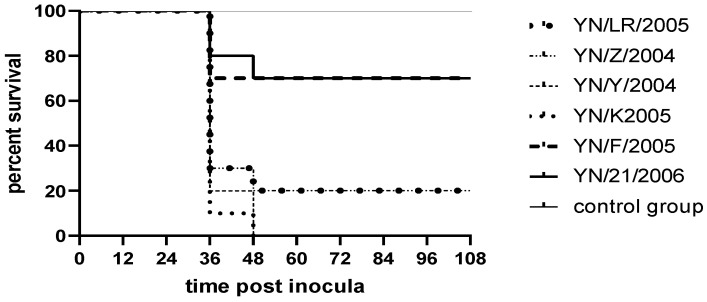
Survival rate results for ducklings.

Following inoculation, ducklings exhibited clinical signs including lethargy, depression, reduced appetite, and, in some cases, ataxia. Deceased ducklings showed opisthotonos (Figure 5). Clinical symptoms appeared as early as 12 h post-inoculation, with mortality occurring within 48 h. Necropsy of deceased individuals revealed hepatomegaly with punctate and brush-like hemorrhages, as well as punctate hemorrhages in the kidneys. In contrast, control ducklings maintained normal mental status and feeding behavior, with no significant pathological changes observed upon necropsy (Figure 6). Survival curves for the infected ducklings were generated using the Kaplan–Meier method and plotted with GraphPad Prism 8 (Figure 7). Most strains (YN/LR/2005, YN/Z/2004, YN/K/2005) exhibited significant differences in pathogenicity toward ducklings and duck embryos (all *p*-values were far less than 0.05) (Table 5).

#### 3.2.3. Cell Adaptation Assay

**Figure 8:**
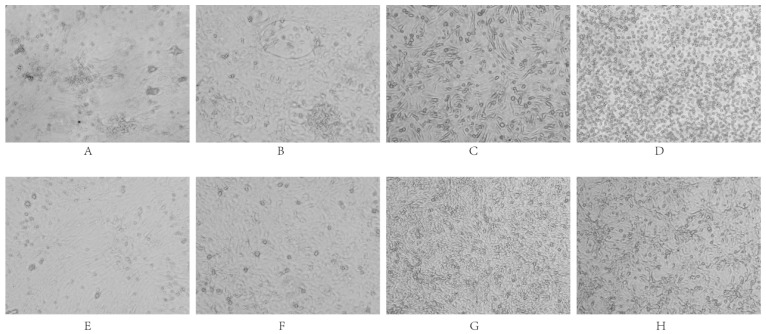
Cytopathic effects of viral infection. DEF cells (Panels (**A**): infected group; (**E**): control group) exhibited clustered infection, followed by filament and network formation, ultimately leading to cell disintegration and death. DEK cells (Panels (**B**): infected; (**F**): control) showed clustered infected cells with widened intercellular spaces; single-layer cells formed filaments and networks before detaching. BHK-21 cells (Panels (**C**): infected; (**G**): control) displayed widened intercellular spaces, cell rounding, and desquamation. Vero cells (Panels (**D**): infected; (**H**): control) demonstrated enlarged intercellular spaces accompanied by numerous rounded cells and desquamation.

The six strains were individually inoculated into DEF, DEK, DEL, DF-1, BHK-21, and Vero cells. After three consecutive blind passages, cytopathic effects (CPE) were monitored. The results indicated that none of the strains replicated efficiently in DF-1 cells, nor did they induce significant CPE. In contrast, all strains exhibited robust replication in DEF, DEK, DEL, BHK-21, and Vero cells, accompanied by varying degrees of CPE (Figure 8).

### 3.3. Genetic Evolutionary Analysis of the YN/LR/2005 Strain

#### 3.3.1. Homology Analysis of the Genome Structures of the YN/LR/2005 Strain

Through analysis of the whole-genome structures of six viral strains, phylogenetic tree construction, nucleotide and amino acid sequence similarity comparisons, combined with pathogenicity assays in duck embryos and ducklings and cell adaptation tests, the YN/LR/2005 strain was selected for in-depth analysis alongside recent reference strains.

Genomic homology analysis revealed that, compared to strains isolated between 2022 and 2024 (Table 6), the YN/LR/2005 strain exhibited nucleotide sequence similarities of 95.5–95.9%, 95.3–95.6%, 96.5–96.9%, and 95.5–95.9% for the whole genome, ORF region, 5′UTR, and 3′UTR, respectively. The nucleotide identities for the structural proteins VP0, VP3, and VP1 ranged from 95.1% to 96.2%, 94.1% to 94.8%, and 95.4% to 96.2%, respectively. In comparison with strains isolated between 2014 and 2018, the YN/LR/2005 strain showed higher nucleotide sequence identities of 96.3–97.3%, 96.2–97.3%, 96.5–97.2%, and 96.8–97.6% for the whole genome, ORF, 5′UTR, and 3′UTR, respectively. The nucleotide similarities for VP0, VP3, and VP1 were 96.0–97.5%, 96.1–97.5%, and 95.4–96.2%, respectively. These results indicate significant divergence in genomic nucleotide sequences between YN/LR/2005 and strains isolated after 2022, whereas sequences show only minor differences compared to strains isolated prior to 2022. Amino acid sequences of the genomic regions remained highly conserved across all compared strains regardless of isolation year.

#### 3.3.2. Prediction of N-Terminal Glycosylation Sites

N-terminal glycosylation sites were predicted using an online software tool. The coding sequence (CDS) of the YN/LR/2005 strain contained 13 potential N-linked glycosylation sites, comparable to circulating strains from 2022 to 2024, among which the WKX03 strain possessed 14 sites. Of these, four sites exhibited high confidence scores, located at positions 288 (NLSN), 457 (NSTS), 592 (NLTS), and 1632 (NVSS). Compared to the 2022–2024 strains, the YN/LR/2005 strain harbored an additional glycosylation site at position 207 (NATD) but lacked the site at position 689 (NQSD). Within the VP1 protein, YN/LR/2005 possessed two potential N-linked glycosylation sites at positions 83 (NGTT) and 98 (NLTS), whereas the recent circulating strains consistently contained an additional site at position 195 (NQSD), absent in YN/LR/2005.

#### 3.3.3. Protein Secondary Structure Prediction

**Figure 9:**
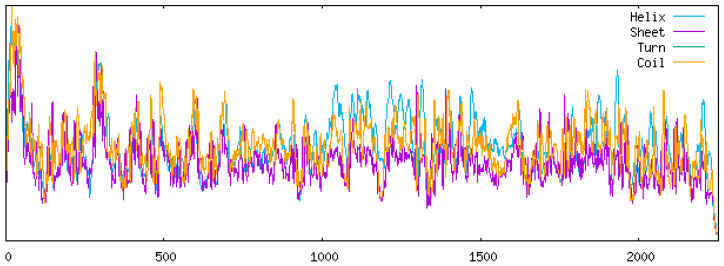
YN/LR/2005 Protein secondary structure prediction diagram.

In the YN/LR/2005 strain, the secondary structure composition comprised 38.16% α-helices, 2.27% β-sheets, 44.20% random coils, and 15.37% extended chains (Figure 9). Among these, random coils constituted the largest proportion, whereas β-sheets represented the smallest (Table 7). No significant differences were observed in the proportions of α-helices and β-sheets between YN/LR/2005 and the DHAV-3 reference strains. However, the reference strains exhibited an increased proportion of disordered coils relative to YN/LR/2005, which may reflect a higher propensity for immune escape mutations in recent viral variants. Concurrently, the decrease in extended chain regions could suggest a reduction in DHAV-3 transmission efficiency in recent years, potentially indicating long-term viral persistence within duck populations. Nevertheless, further comprehensive investigations into viral sequences and biological properties are warranted to validate these hypotheses.

#### 3.3.4. Amino Acid Variation Site Analysis

**Figure 10:**
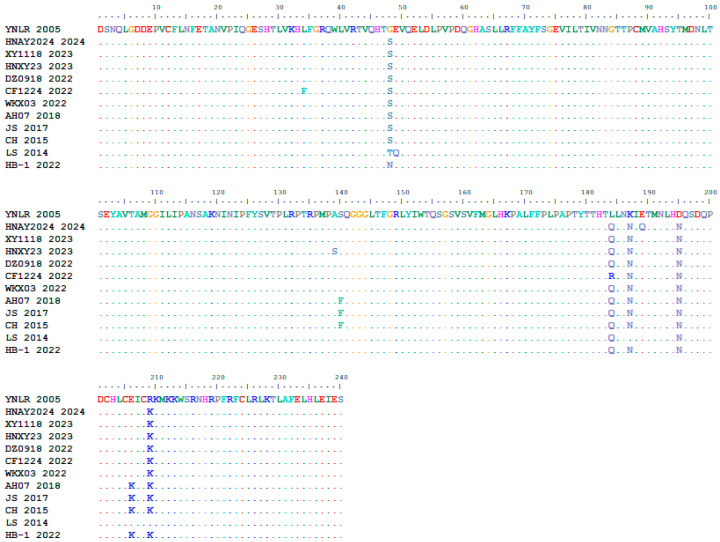
VP1 Amino Acid Multiple Sequence Alignment.

A comparative analysis of amino acid mutation sites between the YN/LR/2005 strain and 11 reference strains revealed a total of 32 unique mutations within the open reading frame (ORF) of YN/LR/2005. Specifically, three mutations were identified in the VP0 region at positions 98 (T→A), 112 (V→A), and 207 (T→N); two mutations in the VP3 region at positions 350 (N→S) and 485 (A→G); five mutations in the VP1 (Figure 10) region at positions 542 (S→G), 678 (Q→L), 681 (N→K), 689 (N→D), and 703 (K→R). In the 2A region, seven mutations were detected at positions 767 (A→V), 774 (G→D), 775 (I→V), 800 (D→G), 900 (S→G), 904 (D→N), and 1024 (A→V). The 2B region exhibited three mutations at positions 1125 (G→E), 1135 (I→F), and 1149 (L→S). Three mutations were found in the 2C region at positions 1248 (I→V), 1253 (N→G), and 1471 (I→V). In the 3A region, three mutations occurred at positions 1549 (S→G), 1553 (H→R), and 1572 (S→T), while a single mutation was observed in the 3B region at position 1588 (T→A). The 3D region contained five mutations at positions 1828 (D→N), 1862 (T→I), 1864 (A→D), 1889 (G→D), and 1927 (S→X) (Table 8). These findings indicate ongoing amino acid variation in DHAV-3, underscoring the need for vigilance regarding potential vaccine escape and the emergence of variant strains with altered host tropism or pathogenicity.

### 3.4. YN/LR/2005 Pathogenicity Experiment

#### 3.4.1. Determination of the Half-Lethal Dose (LD_50_)

The YN/LR/2005 viral stock was serially diluted from 10^−2^ to 10^−8^ to determine the median lethal dose (LD_50_) in duck embryos, ducklings, and the median tissue culture infectious dose (TCID_50_) in DEF cells. The results indicated an embryonic lethal dose 50 (ELD_50_) of 10^−4.5^ per 0.15 mL, a duckling LD_50_ of 10^−2.5^ per 0.5 mL, and a TCID_50_ of 10^−3.25^ per 0.1 mL (Table 9).

#### 3.4.2. Pathological Changes

**Figure 11:**
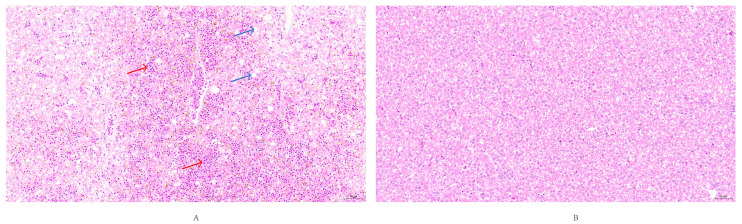
Histopathological lesions in liver tissue (20× magnification). (**A**) Experimental group showing extensive hepatic steatosis (Blue arrow) characterized by numerous round cytoplasmic vacuoles of varying sizes, hemorrhage (Red arrow). (**B**) Control group exhibiting normal liver histology with no observable pathological changes.

Histopathological examination of liver tissues from deceased ducklings revealed extensive lymphocytic infiltration within inflamed areas. Microscopic observation showed erythrocyte rupture due to hemorrhage, accompanied by deposition of golden-yellow pigments. Additionally, pronounced hepatic steatosis was evident, characterized by intracellular vacuolization, reflecting typical hepatic injury. In contrast, liver tissues from the control group exhibited intact cellular architecture with no observable pathological alterations (Figure 11).

## 4. Discussion

Duck hepatitis A virus (DHAV) remains endemic in duck populations across China, posing a substantial threat to the sustainable development of the waterfowl industry [28]; In recent years, DHAV-3 has emerged as the dominant serotype, gradually displacing DHAV-1 as the principal causative agent of duck hepatitis [29]. The considerable genetic diversity and high mutation rates inherent to DHAV-3 raise the likelihood of genetic recombination among circulating strains, which may facilitate viral adaptation and complicate control efforts [7]. This study provides an in-depth investigation of the whole-genome characteristics of DHAV-3, holding significant scientific importance for elucidating its genetic patterns, mutation properties, and prevention and control strategies. Whole-genome evolutionary analysis indicates that the six Yunnan strains isolated in this study exhibit extremely high homology (>99.5%), suggesting they likely originated from a common ancestral virus. These historical strains are most closely related to the 2014 LS strain. Although they share the same evolutionary branch as the most recent circulating strains from 2022 to 2024, they exhibit significant genetic divergence. DHAV-3 nucleic acid sequences (95.3–95.7% homology) exhibited greater variation than amino acid sequences (98.2–98.6% homology). This discrepancy suggests the presence of strong purifying selection pressures during DHAV-3 evolution.

Detailed homology analyses revealed that the YN/LR/2005 strain shares 95.3–95.7% nucleotide identity and 98.2–98.6% amino acid identity with the 2022–2024 reference strains. Prediction of N-linked glycosylation sites within the coding sequences indicated overall conservation between YN/LR/2005 and circulating contemporary strains (except for strain WKX03). Intriguingly, YN/LR/2005 harbors an additional glycosylation site at position 207 (NATD) and lacks one at position 689 (NQSD). The VP1 protein exhibits 195 fewer potential glycosylation sites in YN/LR/2005 compared to recent isolates. These findings align with prior reports describing the relatively conserved nature of DHAV-3 genomes [30]. Of particular interest is the observed increase in irregular coil structures and concurrent decrease in extended chain regions in the reference strains relative to YN/LR/2005, which may reflect adaptive structural modifications potentially enhancing immune evasion or altering viral fitness. Additionally, 32 amino acid substitutions were identified in YN/LR/2005, underscoring ongoing molecular evolution that may influence virulence and transmission. Detailed homology analyses revealed that the YN/LR/2005 strain shares 95.3–95.7% nucleotide identity and 98.2–98.6% amino acid identity with the 2022–2024 reference strains. Prediction of N-linked glycosylation sites within the coding sequences indicated overall conservation between YN/LR/2005 and circulating contemporary strains (except for strain WKX03). Intriguingly, YN/LR/2005 harbors an additional glycosylation site at position 207 (NATD) and lacks one at position 689 (NQSD). The VP1 protein exhibits 195 fewer potential glycosylation sites in YN/LR/2005 compared to recent isolates. These findings align with prior reports describing the relatively conserved nature of DHAV-3 genomes [29]. Of particular interest is the observed increase in irregular coil structures and concurrent decrease in extended chain regions in the reference strains relative to YN/LR/2005, which may reflect adaptive structural modifications potentially enhancing immune evasion or altering viral fitness. Additionally, 32 amino acid substitutions were identified in YN/LR/2005, underscoring ongoing molecular evolution that may influence virulence and transmission.

Consistent with earlier studies, the VP1 gene exhibited hotspot regions of variability, particularly at amino acid residues 57–69, 98–149, and 182–223, with the C-terminal region exhibiting pronounced variability. The segment spanning residues 212–222 encompasses a dominant B-cell epitope critical for host immune recognition [31]. Antigenic domains VP1-c (aa90–175) and VP1-d (aa155–240) are essential for eliciting neutralizing responses, and amino acid substitutions in these regions are implicated in modulating virulence and antigenicity [32,33]. The YN/LR/2005 strain presented five VP1 mutations (S542G, Q678L, N681K, N689D, K703R), four of which localize within the dominant epitope region, Positions 184 and 187 are located within the core region of the neutralizing epitope in DHAV-3’s VP1. Mutations at these sites may alter local surface charge and potentially mask the neutralizing epitope [34]. Position 195 is predicted to be absent from the N-terminal glycosylation potential site (NQSD). Glycosyl displacement has been demonstrated as an immune escape mechanism in multiple viruses, such as influenza and HIV [35,36,37]. The absence of this glycosylation site may alter local protein structure and surface charge, thereby reducing the binding affinity of neutralizing antibodies induced by vaccines based on historical strains. Mutations in these key amino acids could potentially lead to changes in viral antigenicity or virulence, though further validation through subsequent trials is required.

Phenotypic assays revealed divergent cytopathic effects across cell types. Infection of DEK and DEF cells induced classical CPE features, including growth inhibition, cell rounding, detachment, aggregation, filament formation, and intercellular vacuolization, consistent with literature [38,39]. BHK-21 and Vero cells exhibited widespread cell death and intercellular space enlargement, resembling patterns observed in DHAV-1 infections [40]. Conversely, the six isolates failed to replicate efficiently or induce notable CPE in DF-1 cells, and showed limited pathogenic effects in DEL cells, deviating from previous reports of robust CPE in DEL cultures [41]. These discrepancies may arise from strain-specific tropism, passage history, or differential cell susceptibility.

In vivo pathogenicity assessments demonstrated that duck embryos infected with these strains developed systemic hemorrhages and growth retardation, corroborating findings reported by Zhang et al. [42]. Likewise, 7-day-old ducklings manifested clinical signs consistent with DHAV-3 infection, including depression, opisthotonos, and hepatic enlargement with hemorrhagic lesions, in agreement with previous descriptions [43,44]. Histopathological examination revealed brush-like hemorrhages and ecchymoses within hepatic tissue, paralleling observations by Liu et al. [45]. Other studies have reported milder hepatic necrosis and inflammatory infiltration, potentially attributable to differences in duck breed, viral strain virulence, inoculation dose, and host immune status [46,47,48,49]. Emerging genetic variations in DHAV-3 may have contributed to enhanced virulence phenotypes observed in recent isolates, though detailed mechanistic studies are required to substantiate this hypothesis.

This study revealed the genetic evolutionary characteristics of DHAV-3 through genomic analysis of historical strains, but some limitations remain. The hypothesis that VP1 mutations influence antigenicity and immune escape requires further experimental validation. Furthermore, the relatively small sample size may not fully represent the overall diversity of DHAV-3. Expanding surveillance coverage and collecting additional strains from diverse temporal and spatial sources is necessary to construct a comprehensive evolutionary map. Subsequent experiments will validate the specific effects of key VP1 mutations on viral phenotypes and antigenicity through reverse genetics and serum cross-neutralization assays.

## 5. Conclusions

This study conducted comprehensive genomic analysis on six historical strains of DHAV-3 isolated from Yunnan Province. Results indicate these strains share a common ancestral origin but exhibit significant genetic differentiation from strains isolated after 2022, particularly in nucleotide sequence differences within structural genomic regions, while amino acid sequences remain largely conserved. Differences in N-terminal glycosylation sites, alterations in protein secondary structure, and multiple amino acid mutations indicate that DHAV-3 is undergoing continuous evolutionary changes. Long-term systematic molecular surveillance of DHAV-3 is crucial for tracking antigenic drift and providing early warnings about dominant variants that may evade existing immune protection. This surveillance also offers theoretical foundations and molecular targets for vaccine refinement and vaccine strain selection. The five mutations identified in VP1 in this study, particularly those within the immunodominant region, serve as critical genetic markers. They can be used to evaluate the protective efficacy of existing vaccines developed based on historical strains against currently circulating strains, ensuring their antigenicity aligns with prevalent strains. This approach maximizes vaccine protection, enabling effective control of duck viral hepatitis and providing robust support for the healthy development of the waterfowl industry.

## Figures and Tables

**Table 1:** DHAV-3 full-genome amplification primer information table.

Primer Name	Primer Sequences (5′-3′)	Fragment Size
DHAV-3 F1	TTTGAAAGCGGCTGTGGTGTAG	987 bp
DHAV-3 R1	GCTGTATAAAAACATCCAGGAGTGGC	
DHAV-3 F2	GCCCAGGGTGACAACATCTCA	989 bp
DHAV-3 R2	TCTCCATTAGAGACAGGGTACCAT	
DHAV-3 F3	GGCTAACACAGTGACCCCT	1183 bp
DHAV-3 R3	TTCAATTTCCAAATGGAGCTCAAAGGCAA	
DHAV-3 F4	ACTTTTGGCAGGTTGTATATCTGGA	1166 bp
DHAV-3 R4	AAACCCAATGCTACAGCCC	
DHAV-3 F5	GAATCACTTGTTCGTGCCTGT	1264 bp
DHAV-3 R5	TCCAAATACCAAGAGGTTCGGGTC	
DHAV-3 F6	GTGCAAAAAATGTTCAATGGTGGT	990 bp
DHAV-3 R6	ACATTGTGTCCAAATCACTTAGCCTA	
DHAV-3 F7	CCCCTTCAGAAATACAATCATAAAGGT	1054 bp
DHAV-3 R7	TCAGATTTCCTCCAACAAGGGCTA	
DHAV-3 F8	GCACAACATCATGCCAAACA	1144 bp
DHAV-3 R8	GGCACAGACAAAACACAGTCA	
DHAV-3 F9	AGTGACATAGCGTGGAGGG	839 bp
DHAV-3 R9	TTTTTTTTTTTTAGGGTGGGGAGGAATA	

**Table 2:** Genomic information of representative strains used in this study.

Genotype	Strain	Accession Numbers	Country/Year	Length/bp
DHAV-3	HNAY2024	PP977088.1	China/2024	7779
DHAV-3	XY1118	PV125692.1	China/2023	7789
DHAV-3	HNXY23	OR666647.1	China/2023	7779
DHAV-3	DZ0918	PV125691.1	China/2022	7788
DHAV-3	CF1224	PV125690.1	China/2022	7788
DHAV-3	WKX03	PP072258.1	China/2022	7793
DHAV-3	AH07	MT767252.1	China/2018	7786
DHAV-3	JS	MN164467.1	China/2017	7784
DHAV-3	CH	MH752739.1	China/2015	7788
DHAV-3	LS	KP233203.1	China/2014	7792
DHAV-1	WKX01	PP072257.1	China/2021	7709
DHAV-1	FJFZ2020118	MT941533.1	China/2020	7690
DHAV-1	Du	OR738706.1	Egypt/2020	7691
DHAV-2	CUL	OQ862826.1	India/2021	7769
DHV-3	HB-1	OP575303.1	China/2022	7788

**Table 3:** YN/LR/2005 Whole-Genome Structure.

Gene	Location	Gene Length/nt	Amino Acid Length/aa
5′-UTR	1–651	651	/
L+P1	652–2853	2202	734
L+VP0	652–1422	771	257
VP3	1423–2133	711	237
VP1	2134–2853	720	240
P2	2854–5124	2271	757
2A	2854–3768	915	305
2B	3769–4125	357	119
2C	4126–5124	999	333
P3	5125–7407	2283	761
3A	5125–5403	279	93
3B	5404–5505	102	34
3C	5506–6048	543	181
3D	6049–7407	1359	453
3′-UTR	7408–7773	366	/

**Table 4:** Comparison of nucleotide and amino acid sequence homology of DHAV whole genome sequences from different years/%.

Serial Number	Strain	1	2	3	4	5	6	7	8	9	10	11	12	13	14	15	16	17	18	19	20	21
1	YN/F/2005		99.8	99.7	99.7	99.9	99.6	95.7	95.7	95.7	95.7	95.5	95.5	96.8	96.3	96.4	97.3	73.1	73.0	73.1	78.5	95.9
2	YN/LR/2005	99.7		99.7	99.7	99.8	99.6	95.7	95.7	95.7	95.7	95.5	95.5	96.8	96.3	96.4	97.3	73.2	73.0	73.1	78.6	95.9
3	YN/Y/2004	99.8	99.8		99.9	99.7	99.5	95.5	95.5	95.6	95.6	95.4	95.3	96.6	96.2	96.3	97.1	73.2	73.0	73.1	78.5	95.7
4	YN/Z/2004	99.8	99.8	99.9		99.7	99.5	95.6	95.6	95.6	95.7	95.4	95.4	96.6	96.2	96.3	97.2	73.2	73.0	73.1	78.5	95.8
5	YN/K/2005	99.8	99.8	99.8	99.9		99.6	95.7	95.7	95.7	95.7	95.5	95.5	96.8	96.3	96.4	97.3	73.2	73.0	73.1	78.6	95.9
6	YN/21/2006	99.6	99.7	99.7	99.8	99.8		95.5	95.5	95.5	95.6	95.4	95.3	96.6	96.1	96.2	97.1	73.2	73.0	73.0	78.4	95.7
7	HNAY2024 2024	98.3	98.3	98.3	98.4	98.4	98.2		98.5	98.7	98.5	98.3	98.4	97.4	97.1	97.2	94.4	72.7	72.5	72.7	78.0	98.4
8	XY1118 2023	98.4	98.5	98.5	98.6	98.6	98.4	99.6		98.7	98.6	98.4	98.5	97.3	96.9	97.2	94.4	72.8	72.5	72.7	78.0	98.5
9	HNXY23 2023	98.4	98.4	98.4	98.5	98.5	98.4	99.4	99.7		98.9	98.7	98.7	97.5	97.1	97.3	94.4	72.8	72.5	72.7	78.0	98.6
10	DZ0918 2022	98.4	98.5	98.5	98.6	98.6	98.4	99.5	99.7	99.6		99.7	98.6	97.3	97.0	97.1	94.4	72.8	72.4	72.7	78.1	98.4
11	CF1224 2022	98.3	98.3	98.3	98.4	98.4	98.3	99.2	99.5	99.4	99.8		98.4	97.0	96.8	96.9	94.2	72.8	72.4	72.7	78.0	98.2
12	WKX03 2022	98.3	98.3	98.3	98.4	98.4	98.3	99.4	99.6	99.5	99.6	99.3		97.2	96.8	97.0	94.2	72.8	72.4	72.7	77.9	98.4
13	AH07 2018	98.6	98.6	98.6	98.7	98.7	98.6	99.0	99.3	99.2	99.2	99.0	99.1		98.3	98.5	95.4	72.9	72.7	72.9	78.4	97.8
14	JS 2017	98.5	98.6	98.6	98.7	98.7	98.5	99.0	99.3	99.2	99.3	99.1	99.1	99.6		98.7	95.2	72.8	72.7	72.9	78.2	97.4
15	CH 2015	98.5	98.5	98.5	98.6	98.6	98.5	98.9	99.2	99.1	99.2	99.0	99.0	99.5	99.6		95.3	72.8	72.7	72.9	78.2	97.6
16	LS 2014	98.8	98.8	98.8	98.9	98.9	98.8	97.8	98.0	98.0	98.0	97.8	97.9	98.2	98.1	98.1		73	72.9	72.9	78.1	94.7
17	WKX01 2021	83.5	83.6	83.6	83.6	83.6	83.7	83.5	83.5	83.5	83.5	83.4	83.4	83.8	83.7	83.5	83.5		94.7	94.5	72.1	72.9
18	FJFZ2020118 2020	83.5	83.5	83.5	83.5	83.6	83.5	83.4	83.5	83.4	83.5	83.4	83.3	83.8	83.7	83.5	83.4	97.7		95.9	72.2	72.5
19	Du 2020	83.6	83.6	83.6	83.6	83.7	83.6	83.5	83.6	83.5	83.6	83.5	83.5	83.8	83.8	83.6	83.6	98.0	98.0		72.3	72.7
20	CUL 2021	88.8	88.8	88.8	88.8	88.8	88.8	88.1	88.3	88.3	88.4	88.3	88.1	88.7	88.6	88.5	88.5	81.0	81.0	81.2		77.9
21	HB-1 2022	98.3	98.4	98.4	98.4	98.4	98.3	99.4	99.6	99.5	99.5	99.3	99.4	99.2	99.2	99.1	97.9	83.3	83.3	83.4	88.3	

Note: The lower left shows amino acid homology, and the upper right shows nucleotide homology.

**Table 5:** Survival rates of ducklings and embryos with inter-group *p* values.

Strain	Ducklings	Duck Embryos
	Control	
YN/F/2005	0.0671	0.0319
YN/LR/2005	0.0003	<0.0001
YN/Y/2004	<0.0001	0.1483
YN/Z/2004	0.0003	0.0058
YN/K/2005	<0.0001	0.0056
YN/21/2006	0.0669	0.1483

**Table 6:** LR strain homology comparison/%.

Reference Strain	Whole Genome	ORF		5’UTR	3’UTR	VP0		VP3		VP1	
	nt	nt	aa	nt	nt	nt	aa	nt	aa	nt	aa
HNAY2024 2024	95.7	95.5	98.3	96.9	97.6	95.7	98.8	94.1	98.7	95.4	97.5
XY1118 2023	95.7	95.5	98.5	96.5	97.4	95.3	98.8	94.5	99.2	96.2	97.9
HNXY23 2023	95.7	95.5	98.4	96.9	97.6	95.1	98.4	94.7	99.2	95.7	97.5
DZ0918 2022	95.7	95.6	98.5	96.6	97.1	95.7	98.8	94.7	99.2	96.1	97.9
CF1224 2022	95.5	95.3	98.3	96.6	97.4	95.6	98.8	94.7	99.2	95.7	97.5
WKX03 2022	95.5	95.3	98.3	96.9	96.4	95.2	98.8	94.7	99.2	96.2	97.9
HB-1 2022	95.9	95.6	98.4	96.9	96.8	96.2	98.8	94.8	98.7	96.2	97.5
AH07 2018	96.8	96.7	98.6	97.2	97.6	96.5	98.8	96.5	99.2	96.1	97.1
JS 2017	96.3	96.2	98.6	96.9	96.8	96.0	98.8	96.1	98.7	96.0	97.1
CH 2015	96.4	96.4	98.5	96.5	97.1	96.4	98.4	96.2	98.7	95.4	97.1
LS 2014	97.3	97.3	98.8	97.1	96.9	96.7	98.8	97.5	99.6	96.2	98.3

Note: nucleotide-nt; amino acid-aa.

**Table 7:** Analysis of protein secondary structure.

Strain	α-Helix (Hh)	β-Folding (Tt)	Irregular Curling (Cc)	Extension Chain (Ee)
YN/LR/2005	38.16	2.27	44.20	15.37
HNAY2024 2024	38.12	2.71	44.91	14.26
XY1118 2023	37.76	2.13	46.02	14.08
HNXY23 2023	38.34	1.87	46.07	13.73
DZ0918 2022	37.54	2.27	46.29	13.90
CF1224 2022	40.38	1.60	45.18	12.84
WKX03 2022	37.32	1.78	47.40	13.51
HB-1 2022	37.72	2.27	46.02	13.99
AH07 2018	37.67	1.95	45.85	14.53
JS 2017	37.76	2.71	44.82	14.70
CH 2015	37.45	2.27	46.16	14.13
LS 2014	37.89	2.49	44.87	14.75

**Table 8:** Comparison of amino acid mutation sites.

Reference Strain	Major Amino Acid Sites																															
	98 T	112 V	207 T	350 N	485 A	542 S	678 Q	681 N	689 N	703 K	767 A	774 G	775 I	800 D	900 S	904 D	1024 A	1125 G	1135 I	1149 L	1248 I	1253 N	1471 I	1549 S	1553 H	1572 S	1588 T	1828 D	1862 T	1864 A	1889 G	1927 S
YN/LR/2005	A	A	N	S	G	G	L	K	D	R	V	D	V	G	G	N	V	E	F	S	V	G	V	G	R	T	A	N	I	D	D	X
LS 2014		A	N	S	G	T		K		R	V	D	V	G	G		V			S		G	V	G	R	T	A			D	D	
HNAY2024 2024																				I												
XY1118 2023																																
HNXY23 2023			A																													
DZ0918 2022																																
CF1224 2022							R																									
WKX03 2022																																
HB-1 2022						N								N																		
AH07 2018												D	V	G								I		G	R	T						
JS 2017												D	V											G	R	T						
CH 2015				D								D	V											G	R	T						

**Table 9:** Half-Infectious Dose.

Group	Vaccination Dose (mL)	Dilution Ratio							Half-Number Infection
		10^−1^	10^−2^	10^−3^	10^−4^	10^−5^	10^−6^	10^−7^	
duck embryo	0.15	8/8	8/8	8/8	6/8	2/8	0/8	0/8	10^−4.5^
duckling	0.5	6/6	4/6	2/6	0/6	0/6	0/6	0/6	10^−2.5^
DEF	0.1	16/16	14/16	9/16	5/16	2/16	0/16	0/16	10^−3.25^

## Data Availability

The data presented in this study are available in [NCBI GenBank] [https://www.ncbi.nlm.nih.gov/nucleotide/] [PV936567; PV936568; PV936569; PV936570; PV892723; PV892724].
